# World Heart Day — September 29, 2014

**Published:** 2014-09-26

World Heart Day will be observed September 29, 2014. The focus of World Heart Day this year is creating heart-healthy environments in which persons are able to make heart-healthy choices wherever they live, learn, work, and play. Heart disease and stroke are the world’s leading causes of death, claiming an estimated 17.3 million lives in 2008, and representing 30% of all deaths worldwide (1). A heart-healthy environment can help persons make healthy choices to reduce their risk for heart disease. World Heart Day 2014 encourages persons to reduce their risk for cardiovascular disease by promoting smoke-free environments, environments that encourage physical activity, access to healthy food choices, and a heart-healthy planet for all.

CDC is working to help create heart-healthy environments in multiple ways, including community-based approaches, such as the Sodium Reduction in Communities Program (SRCP), and community-clinical linkages, such as the Million Hearts Initiative. SRCP aims to increase access to and accessibility of lower-sodium food options while building the evidence base on population approaches to reduce sodium consumption at the community level. Million Hearts aims to prevent 1 million heart attacks and strokes by 2017 by bringing together communities, health systems, nonprofit organizations, federal agencies, and private-sector partners from across the country to fight heart disease and stroke and their risk factors.

Additional information about World Heart Day is available at http://www.world-heart-federation.org/?id=123. Additional information about Million Hearts, SRCP, and CDC’s Healthy Community Programs is available at http://millionhearts.hhs.gov and http://www.cdc.gov/nccdphp/dch/programs/healthycommunitiesprogram/index.htm.
